# Combination of Size-Exclusion Chromatography and Ultracentrifugation Improves the Proteomic Profiling of Plasma-Derived Small Extracellular Vesicles

**DOI:** 10.1186/s12575-020-00125-5

**Published:** 2020-06-23

**Authors:** Rui Wei, Libo Zhao, Guanyi Kong, Xiang Liu, Shengtao Zhu, Shutian Zhang, Li Min

**Affiliations:** 1 Department of Gastroenterology, Beijing Friendship Hospital, Capital Medical University, National Clinical Research Center for Digestive Disease, Beijing Digestive Disease Center, Beijing Key Laboratory for Precancerous Lesion of Digestive Disease, Beijing, 100050 P. R. China; 2 Echo Biotech Co., Ltd, Beijing, 100010 P. R. China

**Keywords:** Extracellular vesicles, Size-exclusion chromatography, Ultracentrifugation, Proteomics

## Abstract

**Background:**

Circulating small extracellular vesicles (sEVs) and its associated proteins are of great interest in the early detection of many diseases. However, there is no gold standard for plasma sEVs isolation, especially for proteomic profiling which could be largely affected by contamination such as lipoproteins and plasma proteins. Previous studies suggested combinations of different sEVs isolation methods could improve the yield and purity of the isolated fractions. Nevertheless, there is no systematic evaluation of size-exclusion chromatography (SEC), ultracentrifugation (UC), and their combination in a proteomic perspective.

**Results:**

Plasma samples were collected from healthy individuals, and sEVs were separated by one-step SEC, one-step UC, and combining SEC with UC, respectively. Here we exhibited that the purity of sEVs was improved by SEC in contrast to traditional UC. Furthermore, by conducting a SEC procedure followed by UC, we separated sEVs with the highest purity. In the proteomic analysis, 992 protein species were identified in the plasma sEVs isolated by our novel separation method, of which several proteins are sEVs-associated proteins but hitherto never been identified in the previous studies and database, much more than plasma sEVs isolated by UC (453) or SEC (682) alone. As compared to Vesiclepedia and Exocarta databases, plasma sEVs isolated by the new procedure kept 584 previously identified sEVs-associated proteins and 360 other proteins that have not been detected before. Detailed analysis suggested that more kinds of sEVs biomarkers, such as CD9, ALIX, and FLOT1, could be identified in plasma sEVs isolated by the novel isolation method as compared to one-step UC/SEC. Furthermore, the lower abundance ranks of common contaminants, such as lipoproteins and IgG chains, in the sEVs fractions obtained by our new method as compared to one-step UC/SEC also demonstrated the purity of sEVs had been improved.

**Conclusions:**

Combining SEC with UC could significantly improve the performance of mass spectrometry-based proteomic profiling in analyzing plasma-derived sEVs.

## Background

Liquid biopsy is one of the most widely used approaches for non-invasive clinical cancer diagnosis via fast acquiring minimal volumes of body fluids [1]. The human blood sample contains various specific biomarkers, including bioactive lipids, cell-free DNA, mRNA, non-coding RNA, soluble proteins, and small extracellular vesicles (sEVs) [2]. Among all liquid biopsy targets, sEVs attracted much attention during the past decade, which were characterized as nano-sized lipid bilayer vesicles (30–150 nm in diameter) stuffed with RNAs, proteins, and lipids, regardless of their origination [3, 4].

Most biomarker-oriented studies focused on the sEVs-associated nucleic acids, which could be amplified in vitro and easy to detect [5–7]. However, sEVs-associated protein biomarkers are also irreplaceable due to their potential integrability with detecting procedures such as nano-flow cytometry [8, 9]. Nevertheless, the discovery of sEVs-associated protein biomarkers is largely restricted by recent isolation procedures, especially for plasma samples, which contain a dominating pool of impurities such as lipoprotein particles [10, 11]. High levels of plasma lipoproteins (≈10^16^ lipoproteins/mL plasma) are secreted from the liver and intestine, then released and matured in circulation. They are classified into high-density lipoprotein (HDL), intermediate-density lipoprotein (IDL), low-density lipoprotein (LDL), very low-density lipoprotein (VLDL), and chylomicrons (CM), according to their mass and density [12].

Ultracentrifugation (UC) could separate different particles based on a compounding effect of size and density, which is considered as a classic method in sEVs separation [13]. UC is widely adopted in sEVs-associated RNA biomarker discovery, but it is unable to remove abundant lipoprotein particles, such as HDL, which could affect the downstream mass-spectrum analysis of proteins [14].

Size-exclusion chromatography (SEC), also known as “gel filtration”, as an old size-based separation tool, is more and more widely used in the field of sEVs studies after its potential in the separation of sEVs from HDL being full characterized in 2014 [15]. However, SEC also has a limitation. It becomes clear that SEC-based isolation of sEVs can’t remove CM and VLDL, which overlap in size with sEVs.

Theoretically, combining SEC purification with UC enrichment could achieve a considerable isolation efficiency of sEVs with higher purity than a single-step isolation process by removing a large number of HDL, VLDL, and CM. As compared to other combined sEVs isolation methods, in our novel strategy, the biofluid viscosity of plasma samples shows a significant decrease after loaded on the SEC column, so there is no need to dilute samples with 7-fold volume PBS in the next UC process. Thus, combining SEC with UC also helps to simplify the procedures, and operators could deal with a much larger volume of plasma at one time. To fully characterize the combined strategy in sEVs isolation, here we compared it with single-step UC and SEC isolation methods in mass spectrometry (MS)-based proteomic perspective.

## Results

### Combining SEC with UC Successfully Isolated sEVs from Plasma

**Fig. 1:**
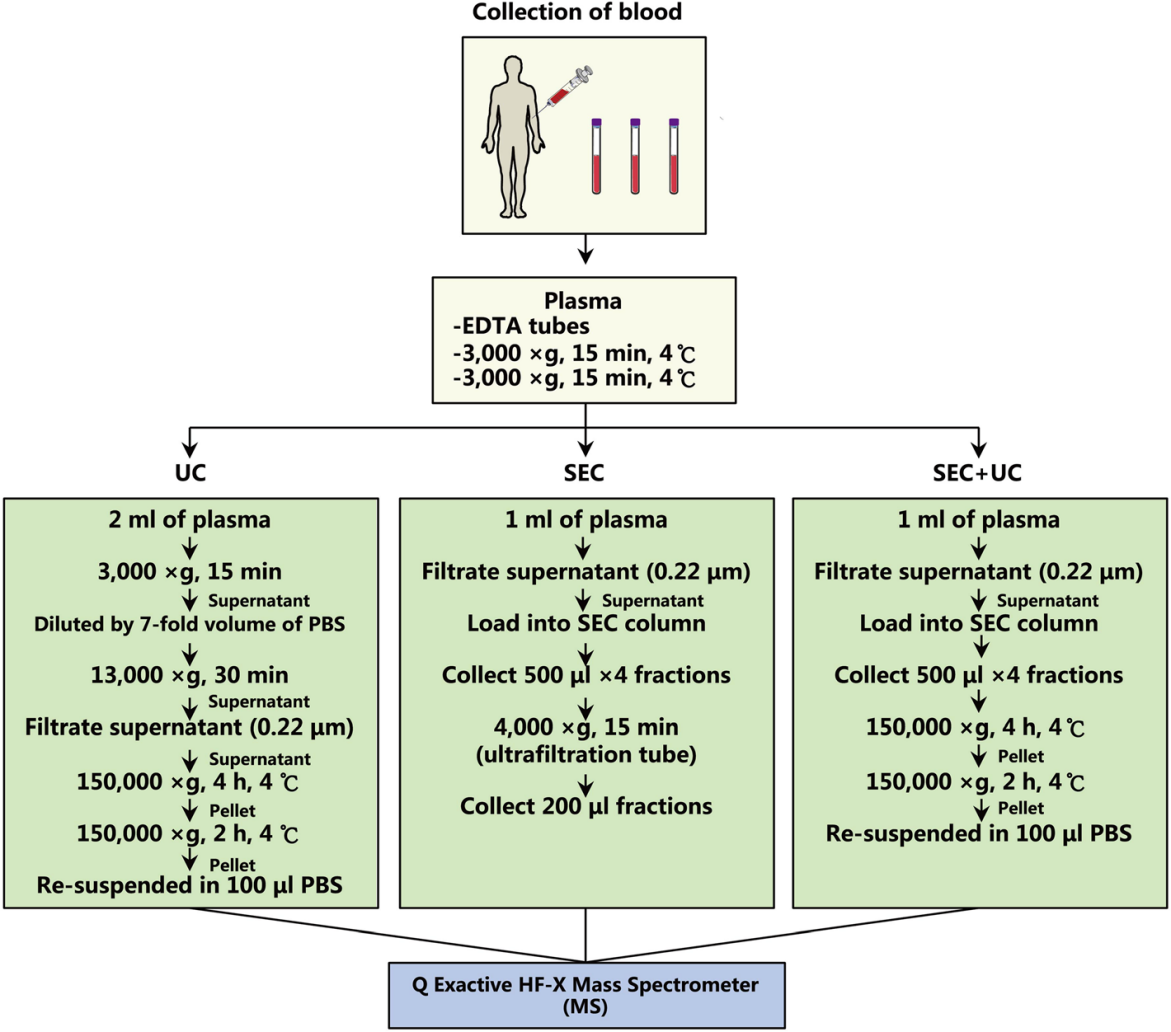
Schematic overview of the experiment pipeline. Blood was collected from three healthy subjects in EDTA tubes, then plasma was isolated. Three isolation approaches were used to separate sEVs from human plasma. Mass spectrometer helped obtain proteomic profiling. *UC* ultracentrifugation, *SEC* size-exclusion chromatography, *SEC + UC* combining size-exclusion chromatography with ultracentrifugation, *MS* mass spectrometer

**Fig. 2:**
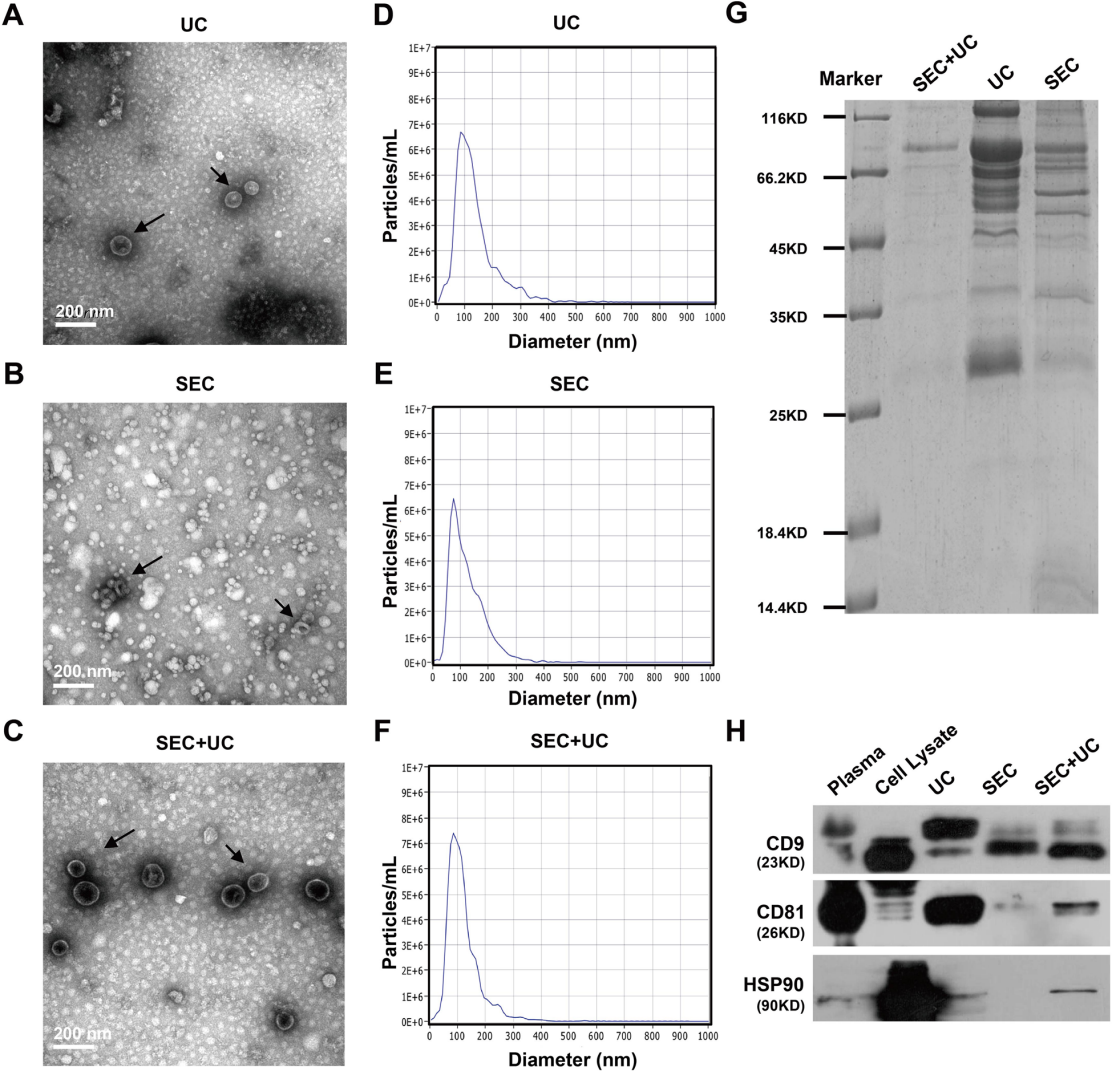
Characterization of plasma sEVs isolated by UC, SEC, combining SEC with UC. **a-c** Transmission electron microscope (TEM) images of sEVs obtained by UC (**a**), SEC (**b**), combining SEC with UC (**c**). All isolated sEVs contained vesicles of variable sizes in the range of 60–100 nm. Black arrows indicate examples of sEVs (cup-shaped). The other white particles were co-isolated lipoproteins. **d-f** The size distribution of sEVs separated by UC (**d**), SEC (**e**), combining SEC with UC (**f**) was measured using nanoparticle tracking analysis (NTA). The images shown were representative of three experiments. **g** Coomassie blue staining exhibited the total protein level of plasma sEVs isolated by three methods. **h** Western blotting showed the expression of three positive markers (CD9, CD81, and HSP90) on the vesicles isolated by UC, SEC, combining SEC with UC

Plasma sEVs were isolated respectively by UC, SEC, combining SEC followed with UC methods, and then characterized according to the MISEV2018 guideline [16]. The operation procedures of the three methods were displayed in detail (Fig. 1). Transmission electron microscope (TEM) images showed that the sEVs isolated from human plasma by three approaches had intact membrane structures and similar morphology (Fig. 2a-c). Then we applied nanoparticle tracking analysis (NTA) to measure the mean diameters of isolated fractions, and most of the particles were at 60–100 nm (Fig. 2d-f). Coomassie blue staining showed the total protein level of separated fractions visually (Fig. 2g), and the pellets isolated by the combination method (SEC + UC) had the minimum total protein content. Subsequently, western blotting confirmed several sEVs positive markers expression (Fig. 2h), including CD9, CD81, and HSP90. Under the premise of the same protein quantity, the fractions separated by the combination method kept all three sEVs positive biomarkers, especially with the highest expression of CD9, revealing the successful sEVs recovery by a combined SEC and UC isolation procedure.

### Combining SEC with UC Successfully Separated sEVs from Lipoproteins and Plasma Proteins with a High Purity

**Fig. 3:**
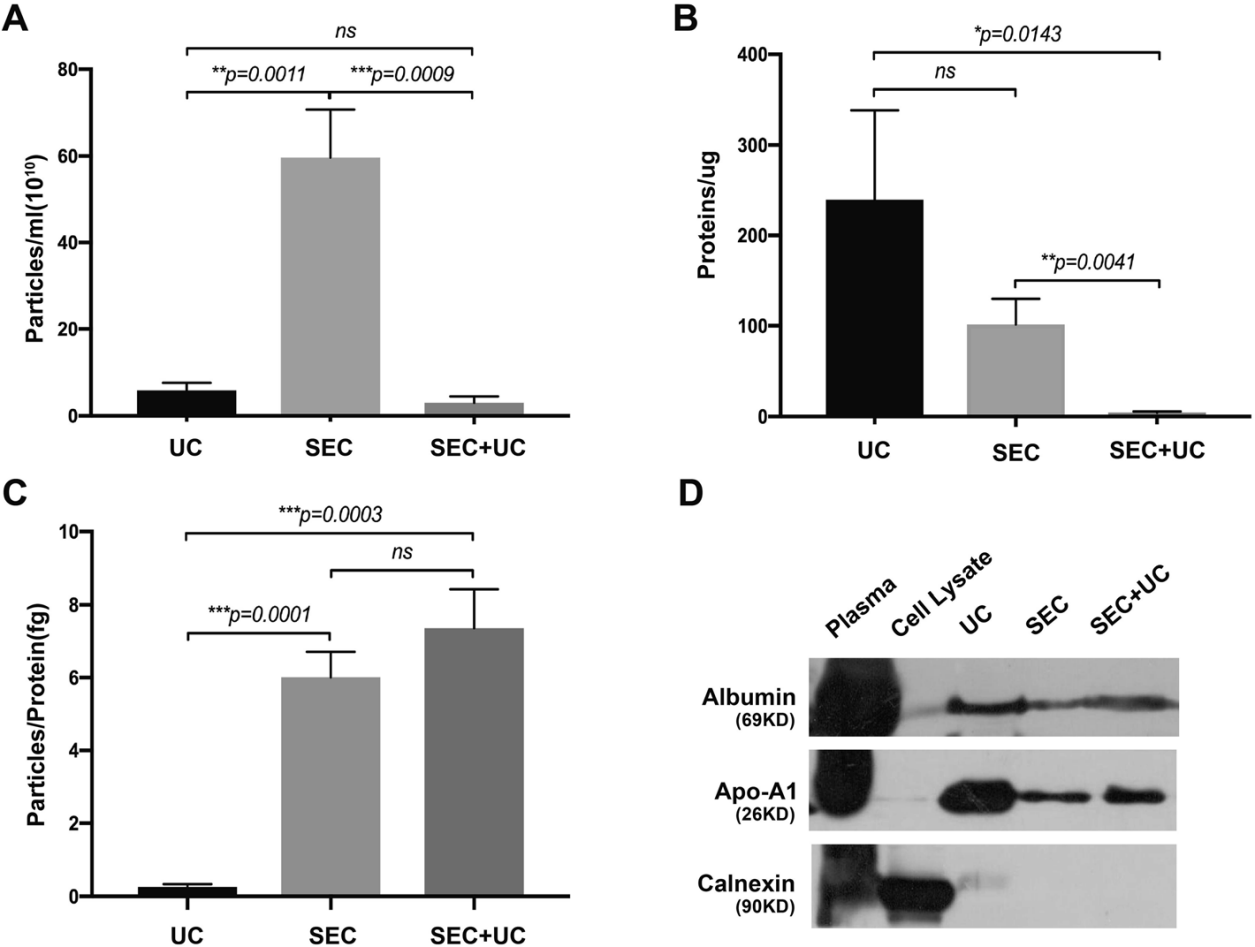
Particle number and protein content of plasma sEVs isolated by UC, SEC, SEC and UC. **a** The particle number of isolated fractions by using UC, SEC, combining SEC and UC was measured by NTA (**p < 0.01, ***p < 0.001; *n* = 3 for each group). **b** The total protein content of isolated particles in three groups was measured by BCA assay (*p < 0.05, **p < 0.01; *n* = 3 for each group). **c** The particle number/fg protein ratio for three groups (***p < 0.001; *n* = 3 for each group). **d** Western blotting showed the expression of two common contaminations (Albumin and Apo-A1) and one negative marker of sEVs (Calnexin) on the vesicles isolated by UC, SEC, combining SEC with UC

Considering direct observation and counting of sEVs (eg. TEM, cryo-EM) is low-throughput and not suitable for quantitative analysis. Indirect assessment of the yield and purity of sEVs (eg. NTA, protein quantification) was widely accepted by the scientific community. According to previously published paper [14], we compared the sEVs yield based on particle number and protein content and evaluated the purity of sEVs by calculating particle number/protein content ratios [17]. According to the NTA quantification results (Fig. 3a), the combining SEC and UC method isolated about 3.03 × 10^10^ particles from 1 mL plasma, evidently less than SEC method (mean = 5.97 × 10^11^/mL plasma, *p = 0.0009*), while no statistical significance was exhibited between the combining method and one-step UC (mean = 5.87 × 10^10^/mL plasma, *p = 0.0984*). BCA protein quantification was performed to evaluate total protein content. The results showed that fractions isolated by the novel strategy had the significantly lowest protein content (mean = 3.99 μg/mL plasma), as compared to UC (mean = 239.55 μg/mL plasma, *p = 0.0143*) and SEC (mean = 101.37 μg/mL plasma, *p = 0.0041*), which suggested a successful removal of lipoproteins and other contaminant particles (Fig. 3b). Considering lipoprotein particles have a higher protein proportion than sEVs, and currently the particle number/protein content ratio is suggested to be an adequate indicator of sEVs purity [17]. In Fig. 3c, our results showed that fractions separated by SEC (mean = 6.01 particles/fg protein, *p = 0.0001*) and the combination method (mean = 7.36 particles/fg protein, *p = 0.0003*) had a better purity of sEVs than that separated by UC (mean = 0.26 particles/fg protein). However, considering NTA cannot distinguish sEVs from similar-sized particles, especially several lipoproteins, the particle number/protein content ratio could only give a rough estimate of sEVs purity, and it is also necessary to validate the recovery rate and purity from other aspects.

Common putative contaminants, such as Albumin and Apolipoprotein A1 (Apo-A1), and one sEVs negative biomarker, Calnexin were all detected in the fractions isolated by UC, SEC, and the combination method (SEC + UC), along with the plasma input. Albumin is the most abundant protein in human blood plasma and considered as the most common pollution indicator. Apo-A1 is a major component of HDL and CM. Western blotting results showed that fractions separated by one-step SEC and the combination method had fewer contaminants, as compared to UC (Fig. 3d).

### More Protein Species Were Detectable in sEVs Isolated by Combining SEC with UC

**Fig. 4:**
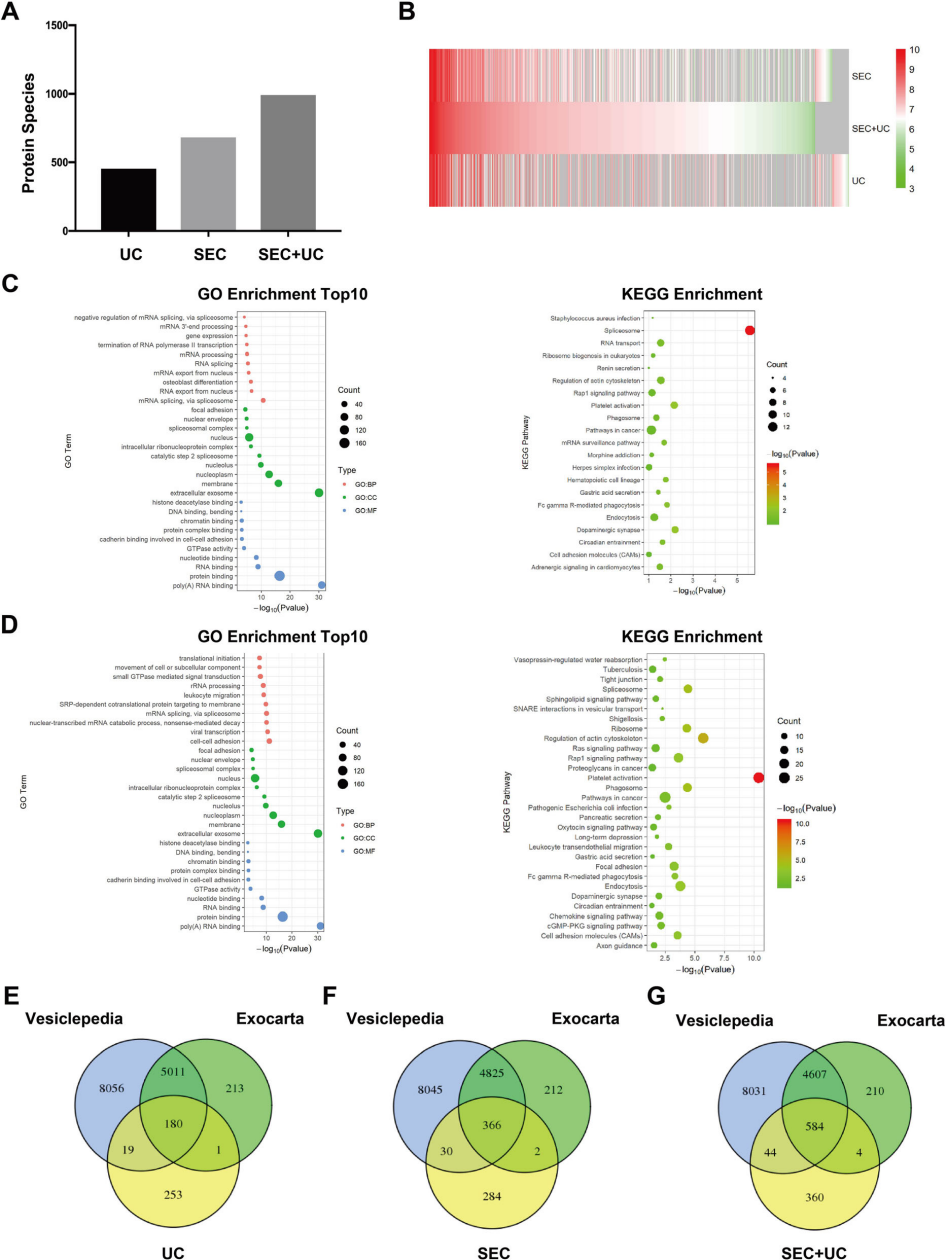
Proteomic analysis of sEVs isolated by UC, SEC, and combining SEC with UC. **a** The total protein species of plasma sEVs isolated by three methods were determined by mass spectrometry-based analysis. UC isolates (protein species: 453), SEC isolates (protein species: 682), combining SEC with UC isolates (protein species: 992). **b** The heatmap showed all detected proteins’ abundance of plasma sEVs isolated by SEC, combining SEC with UC (SEC + UC), and UC. **c** As compared to SEC, there were 345 proteins only identified in the plasma sEVs isolates of the combination method (SEC + UC). GO/KEGG enrichment was carried to analyze these proteins identified by the combination method but not by SEC (left: a bubble plot of GOs; right: a bubble plot of 21 KEGG pathways enriched). **d** As compared to UC, there were 591 proteins only identified in the plasma sEVs isolates of the combination method (SEC + UC). GO/KEGG enrichment was performed to analyze these proteins identified by the combination method but not by UC (left: a bubble plot of GOs; right: a bubble plot of 46 KEGG pathways enriched). **e-g** Bioinformatic analysis exhibited the sEVs-associated proteins species and numbers of plasma sEVs. In total, at the intersecting area of Venn diagram, 180 sEVs-associated proteins were identified by using UC, 366 sEVs-associated proteins by using SEC, and 584 sEVs-associated proteins by using SEC and UC

We continued to analyze the proteins of sEVs fractions by MS. There are 992 protein species identified in the plasma sEVs isolated by combination method (SEC + UC), much higher than those isolated by single-step UC (453) and single-step SEC (682) (Fig. 4a). A heatmap was drawn to exhibit the levels of all detected protein species in sEVs isolated by different methods (Fig. 4b). Moreover, 345 protein species were identified in the sEVs isolated by the combination method but not those isolated by SEC. Five hundred ninety-one protein species were identified in the sEVs isolated by the combination method but not those isolated by UC. Thus, sEVs separated by combining SEC with UC kept much more protein species detectable by MS than single-step UC/SEC isolation.

Then, these 345 proteins identified by combining SEC with UC but not found in one-step SEC were annotated by Gene Ontology (GO) / Kyoto Encyclopedia of Genes and Genomes (KEGG). The enriched GO terms were analyzed into three categories: biological process (BP), cellular component (CC), molecular function (MF). The top 10 enriched GO terms were shown in Fig. 4c, left. For the biological process (BP) category, mRNA splicing via spliceosome (GO:0000398), RNA export from nucleus (GO:0006405), osteoblast differentiation (GO:0001649) were enriched. For the cellular component (CC) category, extracellular exosome (GO:0070062), membrane (GO:0016020), nucleoplasm (GO:0005654) were enriched. For the molecular function (MF) category, poly (A) RNA binding (GO:0044822), protein binding (GO:0005515), RNA binding (GO:0003723) were enriched. Additionally, the KEGG pathway analysis indicated 21 significantly enriched pathways, including spliceosome (hsa03040), dopaminergic synapse (hsa04728), platelet activation (hsa04611) were also enriched in those 345 proteins (Fig. 4c, right).

The same enrichment analyses were performed in the 591 proteins, which identified by combining SEC with UC but not found in one-step UC. The top 10 enriched GO terms were shown in Fig. 4d, left. For BP, cell-cell adhesion (GO:0098609), viral transcription (GO:0019083), nuclear-transcribed mRNA catabolic process, nonsense-mediated decay (GO:0000184) were enriched. For CC, extracellular exosome (GO:0070062), membrane (GO:0016020), focal adhesion (GO:0005925) were enriched. For MF, poly (A) RNA binding (GO:0044822), protein binding (GO:0005515), GTPase activity (GO:0003924) were enriched. Additionally, the KEGG pathway analysis indicated 46 significantly enriched pathways, including platelet activation (hsa04611), regulation of actin cytoskeleton (hsa04810), spliceosome (hsa03040) (Fig. 4d, right).

When we compared our results to the current published public EV datasets, Vesiclepedia and Exocarta. It is clear that there were 584 species of previously identified sEVs-associated proteins (the intersecting area in Venn diagrams) detected in sEVs isolated by the novel approach, much more than protein species of sEVs separated by UC (180) and SEC (366). Additionally, by using this combination method, we identified 360 protein species non-identified and non-reported before in plasma sEVs (Fig. 4e-g). Thus, our results demonstrated that the combination of SEC and UC maintained more protein species to be detectable by MS, including the most previously identified proteins and some novel plasma sEVs proteins, as compared to single-step UC and SEC.

### Combining SEC with UC Kept sEVs-Associated Proteins more Detectable by MS

**Fig. 5:**
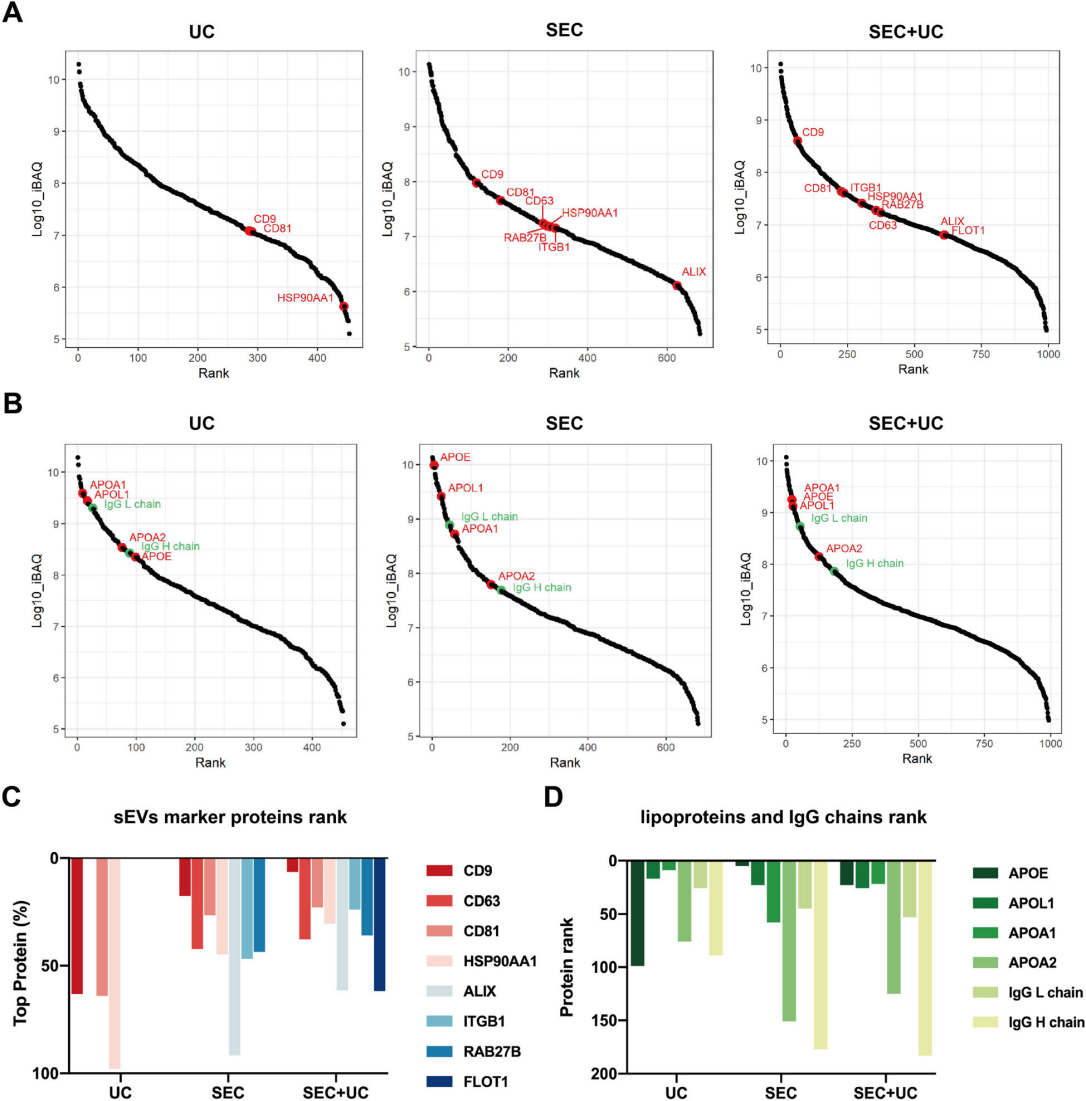
The protein rank of sEVs-associated proteins and common contaminants identified by mass spectrometry. All proteins were ranked by intensity based absolute quantitation (iBAQ) values detected by mass spectrometry, which directly reflected the protein abundance. **a** The diverse rank of sEVs-associated proteins in the isolates of UC, SEC, combining SEC with UC methods. The dot plot highlighted several common sEVs-associated proteins rank, including CD9, CD81, ITGB1, HSP90AA1, RAB27B, CD63, ALIX, and FLOT1. **b** Diverse sEVs contaminants of plasma lipoproteins and other free-floating proteins rank in the isolates of three methods. The dot plot highlighted proteins rank, including APOE, APOL1, APOA1, APOA2, IgG L chain, and IgG H chain. **c** The diagram showed the protein rank of 8 sEVs-associated proteins in total detected proteins among UC, SEC, combining SEC with UC. Top 0% represents the protein expression is the highest in MS-analysis, and Top 100% means the lowest. Data not shown of undetected proteins. **d** The diagram showed the protein rank of 4 lipoproteins and 2 IgG fractions in total detected proteins among UC, SEC, combining SEC with UC. Rank 0 represents the protein expression is the highest in MS-analysis

Then all detected proteins were ranked according to the level of quantified data using intensity based absolute quantitation (iBAQ) values which reflect the abundance value of each protein evaluated by MS. We found that sEVs-associated proteins showed a higher abundance rank in the plasma sEVs isolated by combining SEC with UC as compared to other groups (Fig. 5a, c). Generally, CD9 and CD81 ranked highest among all three groups, suggested them as robust sEVs biomarkers. Flotillin-1 (FLOT1) is considered as a highly specific exosome biomarker, which does not appear in other EVs. Here our results showed that FLOT1 was only detected by MS in the combining group, suggested a better detection ability for exosome components of our new pipeline combining SEC with UC.

Lipoproteins (Apo-E, Apo-L1, Apo-A1, Apo-A2) and IgG antibody fragments (IgG L chain and IgG H chain) are the most common contaminations in plasma sEVs proteomic analysis. All those contaminants in UC group showed a higher abundance rank than the other two groups, except Apo-E, which suggested one-step UC could remove Apo-E efficiently. The high ranked contaminations in SEC group were Apo-E and Apo-L1. As for the novel method combining SEC with UC, a similar protein rank among Apo-E, Apo-L1, and Apo-A1 was observed. As compared to SEC, the combination method showed a better removal effect on Apo-E and two IgG fractions (Fig. 5b, d). Additionally, isolated sEVs from all three pipelines did not contain Argonaute1, Argonaute2, GM130, PMP70, or Tamm-Horsfall protein, suggested that they were not sEVs associated, as previously reported [18]. Moreover, MS analysis also revealed that sEVs isolated by the combination method kept more histones, such as HMGN2, HIST1H1E, H1FX, H1F0, RBBP4, which might be derived from EVs released from cell death (eg. apoptosis, necrosis, NETosis).

## Discussion

Plasma sEVs have attracted much more attention as novel diagnostic biomarkers for cancer, inflammation, and other diseases, since the contained cargoes truly reflect the status of diseased individuals [14, 19]. Currently, more studies are eager to investigate the potential function of proteins contained in plasma sEVs, especially some low-abundant proteins [20]. However, plasma is a complex system and contains a large pool of plasma proteins, such as Albumin, Apo family, which brings great difficulties to the analysis of plasma sEVs derived proteins.

Different isolation methods could influence the purity and yield of plasma sEVs, so there is still no gold method for isolating sEVs with no contaminants. Traditional UC was the most widely used sEVs isolation procedure, with some limitations such as time-consuming, easily influenced by the operator’s experience, and a large abundance of lipoproteins-HDL [21]. The density of HDL is reported to be 1.06–1.21 g/ml, pretty close to the density of sEVs (1.13–1.21 g/ml) [22]. Theoretically, the different particle sizes resulted in a 100-fold faster sedimentation rate of sEVs (100 nm) as compared to HDL (10 nm). However, sEVs are still commonly co-isolated with a significant amount of HDL by UC isolation, for a far more abundant concentration of HDL (10^16^/ml) as compared to sEVs (10^10^/ml) in human plasma [12].

SEC separates sEVs and other particles purely by their size, which could separate particles and small soluble molecules with a diameter smaller or larger than sEVs, such as HDL (5–12 nm), LDL (18–25 nm), and IDL (25–35 nm). Recent studies proved that SEC had a promising potential as it allowed a purer sEVs preparation by removing nearly all HDL particles [23]. However, the molecular sieve effect of SEC filler sepharose CL-2B is incompetent to differentiate particles in the range of 50–100 nm, which showed a poor removal effect for CM and VLDL (30–80 nm). CM is almost the same size as sEVs, but the density of CM is much lower than water. The floating effect of CM, superimposed with the sedimentation effect of sEVs, could well separate CM from sEVs by UC. The density of VLDL is similar to water and could not be subsided by UC. Thus, the UC procedure could separate most CM and VLDL from sEVs, just make up for the deficiency of SEC.

Here we compared the yield and purity of three sEVs isolated assays: one-step UC, one-step SEC and a combination of SEC and UC. By comparing the effects of different methods, we found the combination method was the optimized one. After most of the lipoproteins were filtered out by the SEC column, sEVs were then further isolated and enriched by UC. Our results showed that the purity of plasma sEVs isolated by the novel method is enough for further proteomic analysis. In addition, MS-based analysis revealed that sEVs isolated by the combination assay obtain more kinds of sEVs-associated proteins and some previously non-reported proteins, including some low-abundant proteins.

In the traditional UC, pellets acquired at the final step were washed by resuspension and re-collected by centrifugation, and fewer washing times could reduce the wasting, at the expense of an increased amount of contaminants [24, 25]. Therefore, to achieve a balance between sEVs yield and purity is essential. It is noteworthy that the total particles and total protein content of sEVs we separated by combining SEC with UC were relatively less, while with strong expression of sEVs-associated markers, such as CD9, CD81. Moreover, MS results proved that almost 1000 protein species were identified in sEVs isolated by combination method, higher than traditional UC and one-step SEC, while 360 novel proteins which are not reported before were identified, suggesting this new method showed an improved performance in proteomics profiling. Previous studies used different isolation methods only identified 66–330 protein species associated with sEVs from human plasma by MS. [26, 27] Thus, we exhibited a considerable advantage of combined SEC and UC approach over previous reports in the sample preparation of MS analysis.

Currently, several studies investigated the differential expression of splicing proteins in serum-derived sEVs and ascites, which could be potential biomarkers of early cancer [20, 28], and attractive targets for anti-cancer therapy [29]. Here we sorted out proteins only identified in the combination method, but not in UC, and found that those proteins enriched in many biological processes, such as splicing, transcription, and spliceosome. Thus, plasma sEVs isolated by combining SEC with UC could maintain more kinds of sEVs proteins, as compared to classical UC procedure. Those low-abundant proteins in sEVs isolated by our new approach were not only biologically meaningful, but also promising in disease diagnosis, which would very easily be lost by traditional UC isolation procedures. In general, seeking low-abundant protein is mainly limited by the sensitivity of MS technology and protein degradability [30], and our combined approach could provide an ideal biomarker pool with many low-abundant proteins, further improving the performance of MS.

Previous MS studies suggested density-gradient UC may be a crucial step to further remove more contaminants, but a horizontal rotor with a speed more than 120,000 g and a solvent for suspending sEVs pellets are both needed, which makes the operation tedious. Karimi et al. isolated sEVs by UC + IDC (iodixanol density cushion) + SEC, a more complex procedure than ours, and successfully identified about 1100 proteins, along with contaminants of lipoproteins [2]. Here we used a two-step isolation procedure, conducting SEC followed by UC, and identified almost 1000 proteins. The first step-SEC of our procedure largely decreased the bioliquid viscosity of plasma, which enable the collected fractions to be UC centrifugated directly without any dilution. Therefore, the novel combination method can increase the total amount of plasma processed by the same centrifuge by 7 times, which makes it more time-saving and labor-saving than single-step UC. Generally, we suggested that combining SEC with UC was probably the most cost-efficient sEVs separation method for MS-based proteomics profiling of plasma samples.

## Conclusions

Isolation of sEVs from biological fluids that contain high levels of lipoproteins and plasma proteins is still an arduous task. Here we found that a two-step isolation procedure, SEC followed by UC, could separate sEVs with high purity to make more proteins detectable by MS. Further proteomic analysis indicated that sEVs isolated by the novel method contained not only previously identified sEVs-associated proteins, but also some proteins associated with gene transcription, mRNA splicing, and platelet activation, which were not detected by previous MS analysis of plasma sEVs. These newly identified sEVs-associated proteins could provide a new pool of diagnostic biomarkers for many diseases. Therefore, our study demonstrated the combination of SEC and UC could largely improve the proteomics profiling of plasma sEVs.

## Materials and Methods

The principal aim of this study was to improve the proteomic profiling of sEVs from human plasma, under sEVs acquired with higher yield and purity, helping detect and identify novel biomarkers. To compare the isolation efficacy of three methods (UC, SEC, combination of SEC and UC), NTA and MS were employed.

### Blood Collection and Sample Handling

Blood was donated by three healthy individuals voluntarily. Written informed consent was obtained and this study was approved by the ethics committee of Beijing Friendship Hospital. Peripheral blood samples were collected in EDTA tubes following a regular venipuncture procedure. After centrifugation at 3000×g for 15 min at 4 °C, the supernatant was transferred to new tubes and centrifuged at 3000×g for 15 min at 4 °C again to minimise the platelets contamination.

### Isolation of sEVs by UC

The UC method was optimized according to the procedure previously described [31]. Plasma samples were centrifugated at 3000×g for 15 min. Then, the supernatant was diluted by 7-fold volume of phosphate-buffered saline (PBS), centrifuged at 13,000×g for 30 min, and processed through a 0.22 μm filter. The supernatant was ultracentrifuged using a P50A72–986 rotor (CP100NX; Hitachi, Brea, CA, USA) at 150,000×g, 4 °C for 4 h to pellet the sEVs. The pellet was resuspended in PBS and centrifuged again at 150,000×g 4 °C for 2 h. After PBS washing, the sEVs pellet was re-suspended in 100 μl PBS.

### Isolation of sEVs by SEC

1 mL fresh plasma samples were filtered through a 0.22 μm filter, then the supernatant was loaded into the Sepharose based CL-2B column (Echo9103A-10 ml; ECHO BIOTECH, China) which was prewashed with more than 20 mL sterile PBS in advance. After all samples were into the column and no fluid flowed out from the column bottom, PBS were used to eluate sEVs and other fractions. Each 500 μl of effluent represents one fraction. Then, 4 ~ 7 fractions were collected and added into a 100 KD ultrafiltration tube. After centrifuged at 4000×g for 15 min, the enriched sEVs were collected into a tube for experiments.

### Isolation of sEVs by Combining SEC with UC

Plasma samples were filtered through a 0.22 μm filter and added to the SEC column, as we depicted before. Then 2 mL collected fractions were centrifuged at 150,000×g, 4 °C for 4 h to further pellet the sEVs. The pellet was resuspended in PBS and centrifuged again 150,000×g, 4 °C for 2 h. Finally, the supernatant was removed and resuspended in 100 μl PBS.

### Characterization of Plasma sEVs

The sEVs suspensions were charaterized according to the MISEV2018 guideline [16], the detailed procedures of NTA, TEM, Western blot analysis were performed according to our previous publication [31].

### Protein Quantification by BCA

The protein content of isolates separated by UC, SEC, combining SEC with UC were measured with Pierce™ BCA Protein Assay Kit (Product No. 23,225, Thermo Scientific, USA). 10 μl standard samples and 10 μl sEVs enriched fractions of three methods were pipetted into 96-well plates, then added 200 μl BCA kit to each well. Then the plate was covered and incubated at 37 °C for 30 min. The absorbance was set at 562 nm on the plate reader, and the standard curve was used to measure the protein content of each isolated samples.

### Trypsin Treatment and MS Analysis

Each protein sample was added 3 μL of 1 μg/μL trypsin and 500 μL of 100 mM TEAB buffer and then digested at 37 °C overnight. Equal volume of 1% formic acid was mixed with digested sample and centrifuged at 12000×g for 5 min at room temperature. The supernatant was slowly loaded to the C18 desalting column, washed with 1 mL of washing solution (0.1% formic acid, 4% acetonitrile) for three times, and then eluted twice by 0.4 mL of elution buffer (0.1% formic acid, 75% acetonitrile). The eluents were combined and lyophilized. The lyophilized protein powder was dissolved in 10 μL 0.1% formic acid in water (solvent A), and then injected into a home-made C18 Nano-Trap column (2 cm × 75 μm, 3 μm). Peptides were separated in a home-made analytical column (15 cm × 150 μm, 1.9 μm) with a mobile phrase of 0.1% formic in 80% acetonitrile (solvent B). Sample elution was performed at a flow rate of 600 nL/min by increasing the solvent B concentration from 6 to 100% over 60 min. The separated peptides were analyzed by Q Exactive HF-X mass spectrometer (Thermo Fisher), with ion source of Nanospray Flex™ (ESI), spray voltage of 2.3 kV.

The raw data of MS detection were searched against UniProt database (http://www.uniprot.org). Carbamidomethyl was specified as fixed modifications. Oxidation of methionine and acetylation of the N-terminus were specified as variable modifications. The identified protein contains at least 1 unique peptide with FDR no more than 0.01. GO analysis was conducted, and the databases of Clusters of Orthologous Groups and KEGG were used to annotate the protein family and pathway.

### Statistical Analysis

Statistical tests were performed using R 3.5.1 (www.r-project.org). All tests were two-tailed and False Discovery Rate was controlled for multiple comparisons. *P < 0.05* was considered significant. Packages plyr and reshape2 were used for data sorting and restructuring. VennDiagram, pheatmap, and ggplot2 were used for visualization of results.

## Data Availability

All data generated or analysed during this study are included in this published article.

## Abbreviations

sEVs: Small extracellular vesicles
SEC: Size-exclusion chromatography
UC: Ultracentrifugation
MS: Mass spectrometry
TEM: Transmission electron microscope
NTA: Nanoparticle tracking analysis
HDL: High-density lipoprotein
IDL: Intermediate-density lipoprotein
LDL: Low-density lipoprotein
VLDL: Very low-density lipoprotein
CM: Chylomicrons
Apo-A1: Apolipoprotein A1
GO: Gene Ontology
KEGG: Kyoto Encyclopedia of Genes and Genomes
BP: Biological process
CC: Cellular component
MF: Molecular function
FLOT1: Flotillin-1
IDC: Iodixanol density cushion
PBS: Phosphate-buffered saline
